# Influence of indoor work environments on health, safety, and human rights among migrant sex workers at the Guatemala-Mexico Border: a call for occupational health and safety interventions

**DOI:** 10.1186/s12914-018-0149-3

**Published:** 2018-02-02

**Authors:** Shira M. Goldenberg, Teresita Rocha Jiménez, Kimberly C. Brouwer, Sonia Morales Miranda, Jay G. Silverman

**Affiliations:** 1 Gender and Sexual Health Initiative, British Columbia Centre for Excellence in HIV/AIDS, St. Paul’s Hospital, 608-1081 Burrard Street, Vancouver, BC V6Z 1Y6 Canada; 2 Faculty of Health Sciences, Simon Fraser University, 8888 University Drive, Burnaby, BC V5A 1S6 Canada; 3 Division of Infectious Diseases and Global Public Health, University of California San Diego, 9500 Gilman Drive, 0507, La Jolla, CA 92093-0507 USA; 4 Instituto Mesoamericano para la Gobernanza (IMAG), 53 Calle 42-74, Vista Hermosa IV, Caledonia 4D, Zona 16, Guatemala City, Guatemala

**Keywords:** Sex work, Migration, Migrant sex workers, Work environment, Structural factors, Human rights, Violence, Guatemala, HIV, Sexually transmitted infections

## Abstract

**Background:**

Migrant women are over-represented in the sex industry, and migrant sex workers experience disproportionate health inequities, including those related to health access, HIV and sexually transmitted infections (STIs), and violence. Despite calls for occupational sex work interventions situated in labour rights frameworks, there remains a paucity of evidence pertaining to migrant sex workers’ needs and realities, particularly within Mexico and Central America. This study investigated migrant sex workers’ narratives regarding the ways in which structural features of work environments shape vulnerability and agency related to HIV/STI prevention and violence at the Guatemala-Mexico border.

**Methods:**

Drawing on theoretical perspectives on risk environments and structural determinants of HIV in sex work, we analyzed in-depth interviews, focus groups, and ethnographic fieldwork conducted with 39 migrant sex workers in indoor work environments between 2012 and 2015 in Tecún Umán, Guatemala.

**Results:**

Participant narratives revealed the following intersecting themes to be most closely linked to safety and agency to engage in HIV/STI prevention: *physical features* of indoor work environments (e.g., physical layout of venue, proximity to peers and third parties); social norms and practices for *alcohol use within the workplace*; the existence and nature of *management practices and policies* on health and safety practices; and *economic influences* relating to control over earnings and clients. Across work environments, health and safety were greatly shaped by human rights concerns stemming from *workplace interactions with police, immigration authorities, and health authorities.*

**Conclusions:**

Physical isolation, establishment norms promoting alcohol use, restricted economic agency, and human rights violations related to sex work policies and immigration enforcement were found to exacerbate risks. However, some establishment policies and practices promoted ‘enabling environments’ for health and safety, supporting HIV/STI prevention, economic agency, and protection from violence and exploitation; these practices and policies were especially crucial for recent migrants. Policy reforms and structural workplace interventions tailored to migrant sex workers’ needs are recommended to promote improved working conditions and migrant sex workers’ health, safety, and human rights.

## Background

Approximately half of migrants globally are women, who move across and within national borders for reasons including economic opportunities, family reunification, access to health care, enhanced security, and social mobility [1]. Within destination settings, migrant women are over-represented within precarious and informal employment where they are more likely to face insecurity and unsafe working conditions [2, 3], including the sex industry.

Despite the significant number of women who migrate, prior studies on the health and wellbeing of migrants have primarily emphasized the experiences of male workers [4–6], and limited evidence exists regarding the occupational health and safety of female migrants in destination contexts, including migrant women engaged in sex work. Previous research on migrant sex workers’ health has emphasized vulnerability to HIV, sexually transmitted infections (STIs), barriers to health access, and high levels of violence faced by this population [7–14]. Whereas this research illustrates that migrant sex workers often face different health outcomes and risks than non-migrants [11, 13, 15], few studies have examined structural conditions underpinning these health inequities, including the potential role played by working conditions. Although limited, evidence indicates that migrant sex workers may be more likely to face poor working conditions resulting from intersecting concerns and stressors related to their immigration status, criminalization, human rights violations, social isolation, and dislocation from health and social supports [8].

Whereas quantitative studies indicate that work environments play a critical role in shaping HIV/STI risks among sex workers [16–22], evidence regarding the nuanced and context-specific impacts of specific features of work environments on women’s health and safety remain less well-understood. For example, previous studies have noted differences in HIV/STI risks by work venue (e.g., formal vs. informal establishment, indoor vs. outdoor), yet this relationship varies by context, with sex work in alcohol-serving entertainment venues linked to higher HIV risks in some settings [23–25], while being protective in others [26–32]. Despite mounting interest in workplace interventions for HIV/STI prevention within the sex industry, there remains a critical need to ensure that such interventions are contextually appropriate and grounded in sex workers’ voices and experiences. Importantly, while some previous qualitative research has begun to ‘unpack’ the ways in which work environment features shape HIV/STI prevention for sex workers [33], the perspectives of migrant women remain largely overlooked, particularly within border regions in Mexico and Central America, which are characterized by high population mobility, thriving sex industries, and high rates of violence and human rights violations [34–36].

In light of gaps in knowledge regarding the specific features of work environments and how these relate to the negotiation of sexual risk and safety across different work environments for migrant sex workers, we undertook this qualitative study of migrant sex workers’ narratives to examine the ways in which intersecting features of indoor work environments influence safety and agency to engage in HIV/STI prevention. The analysis was guided by a structural determinants of HIV in sex work framework [37] as well as conceptualizations of structural vulnerability and violence [38–40] which draw attention to the ways in which unequal power and life opportunities shape marginalized women’s health, safety and wellbeing [41]. Our conceptual framework positions sex workers’ health and safety as the product of dynamic and complex structural factors, including intersecting physical, social, and policy features of work environments, which interact with broader macro-structural laws and policies, community organization features, and biological and behavioral factors (e.g., drug use, co-infections) to shape health-related outcomes [37].

## Methods

This study was conducted in the border community of Tecún Umán, Guatemala, located along the main entryway from Central America into Mexico. Intense mobility characterizes this porous border, which hosts a thriving sex industry that attracts a diversity of clients (e.g., agricultural workers, truck drivers) [34, 42–44]. Most sex workers locally are international migrants from other Central American countries or internal Guatemalan migrants [35, 45, 46]. Sex work in this context takes place across diverse establishments, but is primarily concentrated in *formal indoor establishments* such as bars and nightclubs, where sex work is quasi-regulated through public health regulations requiring sex workers within these spaces to receive regular HIV/STI testing and to carry a health permit verifying compliance. Sex work is also practiced in *informal indoor establishments,* including hotels, motels, cantinas (alcohol-serving establishments), and private homes, and to a lesser extent in outdoor settings such as parks, plazas, street corners, and vehicles (e.g., trucks). Sex workers in this region are also exposed to immense risk of violence, and Guatemala has one of the highest murder rates in the world, particularly along the northern border with Mexico [47].

### Data collection

This analysis draws on ethnographic fieldwork (i.e., field observations, informal conversations with key actors), focus groups and in-depth interviews with sex workers in Tecún Umán between November 2012 and February 2015. This research was guided by a Community Advisory Board of sex work, HIV, and women’s organizations and was conducted in close partnership with a local community-based organization dedicated to HIV prevention and education for key populations, including sex workers. All procedures were approved by IRBs at the University of California, San Diego, Universidad del Valle de Guatemala, and the Guatemalan Ministry of Public Health and Social Assistance.

Eligible participants were females≥18 years old, exchanged sex for money, drugs, or other resources in the last 6 months, spoke Spanish, and able to provide informed consent. A community-based team of female outreach workers unobtrusively invited women to participate during outreach to diverse sex work venues. Women were purposively sampled to represent diversity in migration experiences (e.g., internal/international migration), work environments, and age. All participants provided written informed consent, which was designed to maximize participant understanding of procedures and to ensure voluntary participation [48]. Staff guided participants through the informed consent process, explaining the purpose, procedures, and benefits and risks of the study.

Interviews and focus groups were conducted in private offices or the safe/confidential space of women’s choosing. In-depth interviews lasted approximately 1 hour and focus groups lasted 1.5–2 h. The interviews and focus groups followed loosely structured guides that were iteratively revised as data analysis and collection progressed. Discussion topics included sex work and migration histories; working conditions; interactions with police, immigration, and health authorities; violence; HIV/STIs and other health concerns; health access; ethical considerations related to research participation and health access; and recommendations for research and interventions. In-depth interviews were conducted with women who preferred to share their insights one-on-one-setting, and elicited rich data on individual experiences related to our study themes, particularly related to migration, violence, working conditions, and HIV/STIs, and violence amongst migrant sex workers. Focus groups were conducted with women who felt comfortable participating in a group setting and used group interactions to represent different perspectives [49] and generate broader insights [50]. The focus group discussions focused on broader experiences and perspectives related to working across diverse work environments, healthcare access, ethical considerations in research, and recommendations for research and interventions. The interviews and focus groups were conducted concomitantly, allowing the insights gained through each method to inform the other in an iterative fashion.

Participants completed a brief socio-demographic survey and received $10 USD in in-kind goods (e.g., telephone card or household/personal items of their choosing), condoms, HIV/STI prevention information, and referrals to needed medical and social services.

### Data analysis

Given the focus of our analysis on the experiences of migrant sex workers within indoor work environments, the analysis was restricted to the narratives of 39 international and internal migrant sex workers (i.e., moved to Tecún Umán from another city/municipality in Guatemala or another country) who serviced clients in indoor establishments (e.g., bars, cantinas, hotels/motels) [Table 1]. Of the 39 participants included in the analysis, 17 completed individual interviews and 22 participated in focus groups (8 groups).

**Table 1:** Socio-demographic characteristics of migrant sex workers (*N* = 39) in Tecún Umán, Guatemala

Variable	n (%)
Age, in years (median, inter-quartile range)	27 (24–33)
Marital Status	
Single	30 (76.9%)
Married/Common-law	5 (12.8%)
Widow	2 (5.1%)
Divorced/Separated	2 (5.1%)
Education	
None	5 (12.8%)
Primary school or less	21 (53.9%)
Some secondary/finished secondary school	5 (12.8%)
Some prep school or higher	8 (20.5%)
Place of service, last 6 months	
Formal entertainment establishment (bar, casa cerrada)	27 (69.2%)
Informal entertainment establishment (cantina, botanero)	10 (25.6%)
Hotel/motel	7 (17.9%)
Posesses a sex work permit	27 (69.2%)
Country of origin	
Internal Guatemalan migrant	21 (53.8%)
Honduras	9 (23.1%)
El Salvador	3 (7.7%)
Nicaragua	4 (10.3%)
Mexico	2 (5.1%)
Time in current city, in years (median, inter-quartile range)	1 (0.13–4)

NOTE: Data are no. (%) of participants, unless otherwise indicated

Interviews and focus groups were transcribed, translated and accuracy-checked; all identifiers were removed and participants were identified by pseudonyms. Coding was managed in NVivo V.10 [51]. Coding employed a detailed coding scheme that was designed and iteratively revised by SG and TR. Using the constant comparative method [52], coding began with open coding to describe the structure and key emergent themes in the data [48]. As work environment characteristics (e.g., physical attributes, manager interactions) emerged as prominent determinants of vulnerability and prevention of HIV/STI risk and violence, drawing upon concepts of structural vulnerability and structural determinants of sex workers’ health [37], we conducted axial coding to group and regroup the data until a subset of themes emerged which articulated key physical, social, economic, and policy work environment features and their relationship to sex workers’ health and safety.

Our analysis was based on interpretations of data. To ensure rigor, we kept an audit trail documenting key decisions and interpretations related to our analysis; sought analytic consensus among research team members; and compared our data with other research on health inequities, HIV/STIs, violence and working conditions amongst migrant sex workers in other locations. To ensure rigor and the representation of diverse perspectives and experiences within our data, we used a purposive sampling strategy during data collection and throughout the analysis, careful attention was paid to narratives suggesting diverse experiences and differences amongst participants (e.g., those working across diverse indoor and outdoor venues). Furthermore, our analysis explicitly sought to explore both positive and negative impacts of working conditions by examining data that suggested their potential to increase as well as mitigate exposure to sexual risk and violence. Although self-reported data in quantitative study designs are often cited as vulnerable to reporting and recall biases, the insights of migrant sex workers themselves are precisely what we were interested in, which were well illuminated through in-depth interviewing and focus group discussions.

## Results

### Participant characteristics

Of the 39 participants, the median age was 27 (inter-quartile range: 24–33); most participants were single (*n* = 30) and reported low levels of educational attainment, with the majority (*n = 26)* having primary school level education or less (Table 1). Twenty-seven participants serviced clients in formal entertainment establishments (e.g., bars), 17 in informal/unregulated entertainment venues (e.g., cantinas), and 7 in hotels/motels. The majority (69.2%) were registered sex workers. Eighteen international migrants were interviewed, whose countries of origin included Honduras, Nicaragua, El Salvador, and Mexico; the remainder of participants (*n =* 21) were internal migrants from within Guatemala. Among international migrants, over one-third (*n* = 7) were undocumented (i.e., had no legal status in their country of interview).

### Qualitative research findings

Most participants had not previously engaged in sex work prior to their arrival in the border region. Primary drivers of migration described by participants included poverty and family subsistence needs, high levels of gang- and drug-related violence, limited employment opportunities in countries of origin, the need to flee gender-based violence (e.g., childhood abuse, intimate partner violence), and deportation from Mexico or the U.S. Women’s narratives suggested that most had minimal to no exposure to formal HIV/STI prevention prior to their migration and sex work entry. As such, work environments emerged as the most important place where HIV/STI risks and prevention often took place, particularly within the context of migrants’ initial arrival and sex work entry.

Participant narratives revealed the following themes to be most closely linked to their safety and agency to engage in HIV/STI prevention: the impacts of diverse *establishment practices and policies* on condom use and safety; *physical features* of indoor work environments (e.g., physical layout of venue, proximity to peers and third parties); social expectations, norms, and practices for *alcohol use within the workplace*; and *economic influences* relating to sex workers’ agency and control over earnings and clients. Across work environments, women’s health and safety were greatly shaped by concerns stemming from *workplace interactions with government authorities.*

#### Establishment policies for condom use and prevention of violence

Across indoor work environments, establishment owners, managers, and other third parties (e.g., cashiers, hotel managers, janitors) played heterogeneous roles that sometimes supported, and in other cases inhibited, workers’ health and safety. In some formal venues, supportive management policies such as providing information on condom use, security, or regular room checks bolstered agency to negotiate safe sex and deal with violent clients, whereas in other settings, the absence of such protections or outright abusive or exploitative practices by management undermined workers’ health and safety. As recent arrivals, some women had initially begun working in venues where they faced varying degrees of exploitation or mistreatment by management, gradually transitioning to establishments offering greater security and improved working conditions as they gained experience and familiarity with the local sex industry. For example, in describing the first establishments they had worked in, some workers discussed how owners and managers could be exploiters and abusers, whereas others consistently had managers who had looked out for them and their well-being. As one worker described the heterogeneity in managerial practices and policies across indoor workspaces:

There are workplaces where bosses don’t care for their workers. We’re taken care of at the business we work for. The man and the lady take care of us. There are even security cameras to check what they [clients] do upstairs in the rooms.

[Isabel, age 32, bar, internal migrant].

Given most migrant workers’ limited prior exposure to HIV/STI prevention (e.g., condom demonstrations, testing), managers and owners were often key gatekeepers for information on HIV, condom use, and health care access, especially for recent arrivals to the community. Particularly in more formal venues, managers often advised sex workers to use condoms, provided advice or demonstrations on their correct use, or offered onsite condoms. These supports were described as being particularly critical for recent migrants who were new to the community and to the sex industry, and had implications not only for HIV/STI prevention, but also for broader aspects of physical and psychosocial well-being, including workplace violence and work stress:

When I got there, the first thing the lady did was take me to the room…She brought a box of condoms and gave it to me. “Here,” she said. “These are condoms, I don’t know if you’ve seen them before. But you can use this. Each man that you come with should use a condom.” And she came and took one out and explained how to put it on. Because I told her, “You know what, I don’t know. Show me.” So she opened one and took it out and she told me how to use them. .. She’s been very helpful.

[Nayeli, age 22, bar, Honduran migrant].

Women’s narratives also revealed the beneficial impacts of establishment policies to promote security and safety within the workplace, which was most commonly attributed to more formal, higher-end venues such as brothels and nightclubs. Managers or other third parties made efforts to monitor workers’ wellbeing (e.g., room checks) or were willing to intervene in violent situations, such practices were shown to be critical, life-saving interventions. Alejandra shared that she had felt supported by management to deal with an aggressive client. In this circumstance, the client trapped her in the room and attempted to coerce her into unpaid sex; fortunately, she was able to call for help and was supported by the establishment’s cashier and the police to remove this violent client. As several other participants shared:

Once a client told me ‘we are going to do it like this’ [anal]. ‘No,’ I told him and he took out a knife and he wanted to kill me. If the manager wouldn’t have opened the door, he would have killed me.

[Yolanda, age 21, bar, internal migrant].

There are clients who…sometimes say you’ll be half an hour with them. Right, one finishes quickly and leaves. And if one takes a little longer, the boss quickly goes to knock on the door [to check on you].

[Yoselin, age 33, cantina, Honduran migrant].

Other supportive policies employed by some establishments included those intended to promote safety during out-call services, including discouraging out-call visits in circumstances where a worker or client was inebriated; checking client identification prior to out-call visits; and advising workers on the risks of out-call visits:

To take us out [of the bar], they [the clients] had to pay a *caja* [establishment fee] and to pay us for the service and leave their identification in case something happened to us… The other day that the client came for me, he left his identification. And she [the owner] sees that I’m okay and everything is fine.

[Yolanda, age 21, bar, internal migrant].

When they [the managers] see you all drunk they tell you “don’t you take a [out-call] job, you’ve seen what happens, so don’t take [those] jobs”.

[Nayeli, age 22, bar, Honduran migrant].

Despite the efforts of some establishments to provide safer working conditions, participants’ accounts also revealed limitations to the extent to which third parties could protect workers within the broader context of gender-based violence, criminalization, and stigmatization sex workers faced within this setting, suggesting the critical importance of broader structural reforms and community-based efforts to address violence against sex workers. As the following quote suggests:

Right here, a young girl was almost killed two and a half months ago…Right here [at the workplace]…He [the client] almost killed her; he was drugged up and the cashier came and got in a fight with him. So we get some sort of help there.

[Ana, age 36, bar, Nicaraguan migrant].

#### Physical features of indoor workspaces

Participants operating out of formal establishments such as bars typically worked in closer proximity to peers and other potential sources of support. Although working indoors was often linked to enhanced agency to negotiate condoms and reduced exposure to violence, this varied by the physical layout of venues. In most bars, sex work transactions took place in rooms located in the back of venues or upstairs. Where these rooms were more isolated and outside the earshot of managers, cashiers or other sex workers, women expressed concerns regarding their safety and ability to escape violent situations or call for help, whereas transactions conducted in closer proximity to others were reported to facilitate HIV prevention and reduce the risk of violence:

I feel safe when I’m in the *sala* [main bar area]…because the coworkers and the people in charge are there, but it’s hard when one goes to the room and locks themselves [inside] with them [clients] and with the music going on downstairs, you cannot hear anything.

[Carmen, age 26, cantina, Salvadorian migrant].

Across indoor workspaces, threats, assault, or abuse by clients most frequently occurred in the context of refusal to acquiesce to client demands for unprotected or unwanted sexual activities. Although workers typically attempted to negotiate with clients in a more public space (e.g., main bar area), clients often attempted to change the terms of the transaction once they were alone in a private room. As one participant expressed how this contributed to a pervasive sense of fear whenever entering a room with a new client that was out of earshot of managers or co-workers:

I’m scared because when I was working here, one of the girls…went to the room with a client and then when she came out, she was crying…the guy had put a gun to her head if she didn’t perform oral sex. She had to do it because she thought he could kill her inside the room, so those are some of the things that worry us the most, because you never know what kind of person you’re going to bring in [the room]…the place is so big and it’s difficult for anyone to hear you.

[Victoria, age 30, bar, Salvadorian migrant].

Where possible, women took measures into their own hands to mitigate such risks, including leaving a door open or unlocked to be able to call for help, working with peers, servicing regular clients, screening clients in a public area before agreeing to a transaction, and having a weapon available for emergencies. For example, some workers discussed how peers would keep an eye out for one another, especially if a transaction was taking longer than expected. As numerous women described the other security protections they employed:

One time [a client] wanted to do it anal [anal sex] and I didn’t want to because I only work doing it normal [vaginal sex]. He tried grabbing me by force and I got a glass bottle… I hit him in the forehead and I was very scared…he still tried to grab me by the hair, but I was able to escape.

[Carmen, age 26, cantina, Salvadorian migrant].

[I meet clients] here in the park…we would go to a hotel...never in a hidden or an isolated place. In the hotels I can defend myself, there’s people around - the guy in charge, and all that.

[Mercedes, age 26, hotel/motel, Honduran migrant]

Sexual health and safety risks were frequently attributed to working in greater isolation from peers and other sources of support, with workers reporting enhanced stigma, violence, threats, and harassment by clients, police, and the community when soliciting in more isolated spaces. While working more independently (e.g., in hotels vs. bars) was felt to offer some benefits including greater autonomy, women also felt these settings placed them in more vulnerable situations due to the limited protections they offered. As one participant explained, in such circumstances, “we have to manage ourselves the best we can.” For similar reasons, participants ascribed particular importance to avoiding out-call visits, although this was most common among those working in formal settings such as bars. Many women recounted instances of violence, disappearances, or homicides occurring within the context of out-call visits, and as such, described undertaking measures such as avoiding leaving with unknown clients or only servicing clients within their primary work venue as a harm reduction strategy. As a young Honduran migrant explained:

There are some that do leave [with a client], but I never go. I’m always thinking of my kids because in the last few months one of my coworkers recently disappeared. She went to go work somewhere else and when she came back she was dead. Supposedly they paid her 800Q [approx. $100 USD] to leave [out-call] and they went out looking for her and she never came back until she appeared on the other side [of the border].

[Carmen, age 26, cantina, Salvadorian migrant].

#### “If you don’t drink, there’s no money”: Substance use as a barrier to condom negotiation and safety

In alcohol-serving establishments, migrant women often described social and economic pressures for alcohol use during sex work as undermining their capacity to negotiate safer sexual practices with clients. In formal venues such as bars, women are expected to *fichear* (i.e., drink and flirt with clients) prior to negotiating transactions and receive a commission for each beverage purchased for them by clients, with a larger ‘tip’ for alcoholic vs. non-alcoholic beverages. Due to the gendered nature of substance use and taboos against female alcohol use, particularly outside the context of sex work and in smaller communities, most women hadn’t typically drunk alcohol prior to migrating to the border region and beginning to engage in sex work, and described beginning to do so as an economic necessity related to their sex work:

Q: At what age did you start drinking and why?

A: When I started this [sex work]…it had to be done…they don’t demand it but you practically have to do it because if you don’t drink, there’s no money.

[Rocio, age 31, bar, Honduran migrant].

Whereas many recognized the risks of excessive alcohol use to their health and safety while working and tried to avoid or reduce their alcohol use, they also acknowledged how economic pressures and social norms for alcohol use within alcohol-serving establishments made it difficult to do so:

I drink, but not a lot. Sometimes I drink a lot because someone buys for me or because I’m *fichando* [drinking with/entertaining clients]. I’m making money - it’s not because it’s my dream to be drinking.

[Nayeli, age 22, bar, Honduran migrant].

Women held serious concerns regarding client violence and the risks and challenges of attempting to negotiate safer sex with intoxicated clients. Almost all participants described circumstances in which they had experienced violence or threats (e.g., verbal or with a weapon) within the context of clients’ substance use. As one bar-based worker put it, “When they’re [clients] drunk or armed or drugged up, there are men who are really crazy.”

Such concerns regarding client intoxication intersected with physical attributes of work environments, as sexual assault, physical violence, and unprotected sex were all felt to be much more likely to occur in settings where women would be unable to call for help in circumstances where intoxicated clients posed a risk to their health or safety:

When I go to work…I start feeling uncomfortable. Being there and someone might want to abuse you…I’m afraid that somebody will hit me when I say I will protect myself [with a condom], and when they’re inside [the room], since they’ve already been drinking they don’t want to [use a condom] anymore, by then they just want to hit someone. That’s the fear of entering the room.

[Carmen, age 26, cantina, Salvadorian migrant].

The violence happens once people are drunk…clients that humiliate you inside the room. They start saying that if they don’t get what they want, they want their money back…some try to take off the condom or carry weapons or knifes, so we have to withstand the humiliation. For instance, the room is here, but the business [main bar area] is over there, so when you go into the room you’re by yourself and they could just leave you there dead or alive, while nobody is going to find out until later.

[María, age 44, bar, internal migrant].

Although expectations for alcohol use within alcohol-serving establishments typically included implicit or explicit expectations to drink with clients, establishment practices and norms related to alcohol use varied across workspaces. For example, some establishments implemented harm reduction approaches including removing inebriated clients from the premises or by helping workers avoid intoxication, such as by providing them with watered-down drinks or by monitoring their alcohol use, whereas other venues took a more ‘hands-off’ approach. As Yolanda explained:

Well, it’s good [at my current workplace], because it doesn’t require you to drink…they take care of you, they don’t let you to go around drinking or walking around being a disaster. Because there are other places where things happen and that [being drunk] can hurt you.

[Yoselin, age 33, cantina, Honduran migrant].

#### Variations in economic pressure, agency and autonomy across work environments

Migrant women’s narratives revealed the importance of balancing competing priorities in their lives, including those related to their economic needs as well as the need for security and safety at work. Most women had children and worked primarily to meet their subsistence needs and to send remittances to family members in home communities.

Women’s descriptions of economic pressures and agency within indoor work venues largely depended on management polices and practices. In more formal establishments, a cashier typically collects workers’ earnings directly from clients, subtracts establishment fees (e.g., room fee per client, extra fees for out-call visits), and pays workers the remainder as salary. In some venues, women receive tokens rather than cash as payment, which some workers felt was beneficial in terms of protection against robbery by clients or other workers:

We can easily leave the money there with the cashier and they return the money to us a week later. They give us a token that identifies us if it’s for percentages, if it’s per *ocupada* [transaction], how much it costs and they make their calculations and give us our money…They don’t steal our money like in other places.

[Carmen, age 26, cantina, Salvadorian migrant].

Alternatively, women operating in more informal venues (e.g., hotel/motels) perceived this as enhancing economic independence, agency and control due to their ability to negotiate prices and transactions on their terms, select their own clients and schedule, and keep their full earnings from sex work. As Francisca explained:

I don’t like to deal much with intermediaries because they ask for some amount [of our earnings] and they give a certain amount to us and they take another amount for them. That way is not profitable for us…There are people here who recruit, men and women who find clients for us, but their business is to say to us “you will need to pay me this amount”…So, for me it’s better to have an independent business.

[Francisca, age 39, hotel, internal migrant].

Although working in more formal establishments was often felt to offer some important health and safety benefits, women also expressed concerns regarding what they perceived to be inequitable payment structures within these venues. In some bars, women doubted that they were being paid their agreed-upon earnings, and feeling cheated out of their earnings was most commonly reported during migrants’ initial arrival and sex work entry. In many bars, women also described policies where their pay was reduced in cases where they leave the workplace without the manager’s permission or were late for work. Concerns regarding unjust payment arrangements were also more likely to be experienced by more recent arrivals and by women who lived in the bar where they worked, for whom these circumstances could result in high debts and a lack of control over their earnings.

Women operating out of informal settings often positioned their working conditions as a ‘lesser of evils,’ acknowledging the trade-offs they made between their desire for enhanced economic agency and the enhanced physical and psychosocial vulnerabilities arising from working independently, such as experiences of violence and trauma, social isolation, and economic insecurity. Many informal workers described initially working in formal establishments during their arrival and entry into the sex industry, and eventually shifting to more independent forms of work due to these perceived benefits:

Some time ago, when I was a girl, I worked at an establishment, but after that I haven’t gotten involved in a business directly, besides they exploit us and they don’t pay us what we should be paid, or they don’t pay what was agreed, they take money from you who knows what for…that’s why I work on my own. I know the little money I’m going to make, but I know that it will be mine… Another thing is that one risks one’s life on the streets due to the problems with men, nobody gives a damn about us. I work on my own; I don’t work so that somebody else bosses me around.

[Celeste, age 39, cantina, Nicaraguan migrant].

#### Workplace interactions with police, immigration authorities, and health inspectors

Across work environments, migrant sex workers’ safety and HIV/STI prevention capacities were strongly shaped by interactions with police, immigration authorities, and government health inspectors. Rather than being perceived as sources of protection, government officials such as police and immigration authorities were often positioned as participants’ greatest source of stress and fear, particularly for international and undocumented migrants fearing deportation or other negative immigration consequences.

The nature and consequences of migrant sex workers’ experiences with government authorities varied across work venues. While sex work remains criminalized in Guatemala, in Tecún Umán, as in many other Latin American cities, public health regulations require sex workers within formal establishments (e.g., bars) to maintain a permit demonstrating routine HIV/STI testing at the municipal health clinic. Although women prioritized the importance of voluntary HIV/STI testing, they were critical of the punitive nature of these regulations, which were often used as a basis for authorities to abuse and punish sex workers [53]. As such, women often noted that compliance with these public health regulations served as an important means of avoiding arrest, detention or deportation. As one participant explained, “When the police drops by…if you don’t have the stamp with the date when you last went to the Health Department for your checkup, that’s where the problem begins.”

During the course of our fieldwork, we observed and frequently heard accounts of the increasing intensity of raids and inspections by police, health inspectors, and other government authorities on indoor venues in Tecún Umán as well as other nearby cities (e.g., Quetzaltenango, Escuintla, Guatemala City). During such raids, government officials were often implicated as either direct or indirect perpetrators of violence and human rights violations against sex workers, with migrants with precarious legal status (e.g., undocumented) reporting enhanced vulnerability to such abuses. In bars and cantinas, the consequences of raids often depended on the management style and relationships between management and government authorities. In some circumstances, establishment managers and owners provided workers with protection from authorities to the best of their abilities (e.g., by notifying them of upcoming raids). Workers in informal establishments such as cantinas described this as particularly critical, given that public health regulations prohibit sex work within such spaces:

They [managers/owners] talk to each other, “look, there’s a raid right now, the police are over there, taking women from the cantinas”…the boss tells us to go take a walk and then come back. But I’ve never had bad luck… [otherwise] they would take me and deport me.

[Yoselin, age 33, cantina, Honduran migrant].

They [managers/owners] always let us know that there will be a raid and what we do is leave or the manager will close the bar. He will close the bar if it’s during the night or day.

[Carmen, age 26, cantina, Salvadorian migrant].

However, in other venues, rather than providing protection from authorities, some establishments worked in complicity with corrupt officials in ways that enhanced workers structural vulnerability. In some cases, workers described human rights violations and enhanced health risks they faced as a result of authorities’ abuse of power and pressure by establishment managers to acquiesce to the demands of officials seeking to abuse or extort workers. In one case, Ana described being pressured by her manager to have unpaid sex with a police officer as a means of the venue avoiding prosecution for having underage workers present:

She [the manager] said, “I’m going to have problems [with the police] because you don’t want to go with him”. So she was kind of forcing me…the lady was kind of upset with me, because I didn’t go… Others would tell me that what the lady would do is if a policeman liked you, you would have to ‘pay’ for the others. So you would have to…sleep with the police for free. "Excuse me? I don’t do that," [I said]. There were some that would hide because they said that there were policemen that, they had to go with them.

[Nayeli, age 22, bar, Honduran migrant].

Workers often discussed how intersecting vulnerabilities related to stigma, criminalization, xenophobia, and their immigration status resulted in a lack of access to justice and increased vulnerability to workplace exploitation. International migrants were particularly fearful of interacting with authorities, and most felt that they did not have anywhere to report violence or other crimes committed against them, which some establishments took advantage of to exploit migrant workers. Women’s testimonials revealed how their status as both migrants and sex workers rendered them particularly vulnerable to exploitation by establishment owners and government authorities, particularly as recent arrivals to Guatemala:

It’s the fear you feel being a foreigner…I’ve worked in business like that; because one is are Guatemalan, and that is a problem, they say, we’re going to call immigration…to intimidate us…to make us scared. Because it happened to me that I worked in a business and the owner didn’t pay me and she said to me, “no, no, you aren’t going to do anything because you aren’t from here. I have the police on my side.” So I left. And as much that I worked, she didn’t pay me. Because I’m not from here, the police from over there, they didn’t do anything.

[Celeste, age 39, cantina, Nicaraguan migrant].

## Discussion

In this study, migrant sex workers’ narratives suggested that safety and agency to engage in HIV/STI prevention was strongly shaped by intersecting physical, social, economic, and policy features of indoor establishments. Across work environments, participants highlighted the extent to which their safety and HIV/STI prevention capacities largely depended on structural conditions related to their work environment, suggesting the critical need for occupational health interventions targeting recent migrant sex workers. Physical isolation, establishment norms promoting alcohol use, restricted economic agency, and human rights violations stemming from the implementation of sex work and immigration policies exacerbated risks, whereas establishment policies and practices that supported HIV/STI prevention, economic agency, and provided protection from police and immigration authorities promoted an ‘enabling environment’ for workplace health and safety. These findings are supported by a recent qualitative review and meta-synthesis of work environments and HIV/STI prevention, which found that establishment policies and practices that supported occupational health and safety, protective practices of third parties (e.g., condom promotion), and the ability to work with peers represent critical ways of enhancing safety and sexual risk negotiation within indoor work environments [33]. These findings support calls for safer work environment interventions, which that have been shown to positively impact the health of marginalized groups [54].

While there remains a critical need to ensure that local, national, and global HIV/STI prevention policies and programs are rooted in the voices of key affected populations, most research on sex work environments has focused on the experiences of non-migrant women [33]. This study provides unique insight into the ways in which concerns regarding migrant status (e.g., social isolation, newcomer status, legal status) intersect with features of work environments (e.g., managerial policies and practices, alcohol use, physical features of ‘place’) to shape migrant sex workers’ structural vulnerability and resilience to HIV/STIs, violence and human rights violations. While punitive enforcement-based responses to sex work and immigration exacerbated health and social inequities for recent and international migrants, the protective role played by some managers was particularly vital for recent migrants, who often arrived in destination settings with no prior exposure to HIV/STI prevention, substance use, or sex work, and relied heavily on their workplace for information and resources regarding these issues. As recent arrivals often faced the greatest concerns regarding exploitation and the consequences of interacting with police, health, and immigration authorities, where managers made efforts to protect workers from human rights abuses (as opposed to engaging in exploitative practices themselves), this was crucial for reducing susceptibility to punitive consequences such as deportation, detention, or extortion.

Whereas prior research has tended to emphasize primarily risky, rather than health-promoting, features of sex work environments [8], this study documented various resilience-based and health-promoting practices implemented by sex workers (e.g., working with peers), as well as managers, owners, and other third parties within indoor spaces. These findings are supported by evidence from other settings highlighting the diversity and importance of protective practices of third parties for sex workers’ health and wellbeing. Qualitative studies from India, Canada, Cambodia and China have identified the protective role of working in establishments where HIV prevention and education is promoted (e.g., provision of condoms at work, managers discussing and establishing norms for condom use), insofar as such practices respect workers’ wellbeing, agency and human rights, as opposed to taking a more punitive approach [33, 55–58]. Our findings regarding the potentially protective role managers can play in mitigating negative interactions with authorities are supported by findings of the *Sonagachi* project in India, where sex workers reported improved relations with police at an establishment level (e.g., managers and peer engagement to stand up against unjust police practices) [55, 57].

Our findings indicate the critical need for occupational health and safety interventions particularly directed at recent and undocumented migrants, who may otherwise lack access to HIV/STI prevention and information regarding workplace health and safety. Although working in supportive workspaces has been shown to be important for supporting sex workers’ safety and mitigating HIV/STI risks [33, 59], such models remain severely restricted within contexts characterized by criminalization, violence, and human rights abuses against sex workers. While occupational interventions that build upon existing models of supportive indoor workspaces are clearly warranted, these must take place alongside broader policy reforms, including the removal of punitive and criminalized approaches that restrict migrants and sex workers’ rights [9, 16]. The unique health, safety, and economic vulnerabilities reported within informal venues also largely stem from structural criminalization, violence, and stigma surrounding sex work, and policy reforms to promote access to safe, voluntary, and non-exploitative workspaces remain needed.

This study has several limitations. Although our qualitative study design does not permit direct generalization of findings to a wider population, the insights offered by this study provide key contextual data regarding the nuanced and intersecting impacts of indoor work environment features on migrant sex workers’ health and safety, which are not well-captured by traditional epidemiologic designs. Given previous research on street-based work environments and gaps in knowledge regarding the specific features of indoor work environments that confer resilience versus vulnerability [18, 33, 60, 61], this study focused on indoor environments only. Future mixed-methods research is recommended across diverse contexts and populations to develop evidence-based interventions to promote safer work environments for migrant and non-migrant sex workers in Latin America and elsewhere. Finally, given the alarmingly common nature of workplace violence and other human rights abuses reported by our participants, further research and interventions that address intersecting experiences of trauma, mental health, and substance use in relation to health and social inequities faced by migrant sex workers and other marginalized populations of women remain crucial areas for further research.

## Conclusions

In this study, migrant sex workers’ safety and agency to engage in HIV/STI prevention was strongly shaped by intersecting physical, social, economic, and policy features of indoor establishments. Physical isolation, establishment norms promoting alcohol use, restricted economic agency, and human rights violations perpetrated by authorities exacerbated risks, whereas managerial practices that supported HIV/STI prevention, enhanced security, and provided protection from police and immigration authorities promoted an ‘enabling environment’ for migrant sex workers’ wellbeing. Policy reforms and workplace interventions that build upon existing supportive practices of managers and peers are recommended, with a particular need for strategies tailored to recent migrants’ needs.
